# Exploring Gender Differences in Early Weight Change and Variability in Adolescents with Anorexia Nervosa during Inpatient Treatment

**DOI:** 10.3390/jcm13113255

**Published:** 2024-05-31

**Authors:** Georg Halbeisen, Karsten Braks, Thomas J. Huber, Georgios Paslakis

**Affiliations:** 1 University Clinic for Psychosomatic Medicine and Psychotherapy, Medical Faculty, Campus East-Westphalia, Ruhr-University Bochum, 32123 Luebbecke, Germany; georgios.paslakis@rub.de; 2 Centre for Eating Disorders, Klinik am Korso, 32545 Bad Oeynhausen, Germany

**Keywords:** eating disorders, anorexia nervosa, bulimia nervosa, binge-eating disorder, adolescents, psychotherapy, treatment outcomes

## Abstract

**Background:** Adolescents’ early responses and overall outcomes during anorexia nervosa (AN) treatment may differ by patient gender, raising the question of whether evaluating clinical data during AN treatment may require different criteria. **Methods:** We investigated, using patient records, whether young men and young women with AN differed in terms of early treatment response (defined as weight change and variability within the first 14 days) and whether early treatment responses predicted treatment outcomes similarly across genders. **Results**: Weight changes predicted patient discharge weight across all gender groups. Weight variability predicted higher disordered eating psychopathology and higher body image insecurities at discharge. Gender differences emerged only for weight gain, which was more pronounced for young men, and gender modulated the effects of weight gain and variability on general psychopathology outcomes. **Conclusions:** The present findings suggest that early weight changes and weight variability are similarly important predictors of AN treatment outcomes in adolescents but also hint at possible gender differences in terms of the link between weight change and, respectively, variability on general psychopathology.

## 1. Introduction

Anorexia nervosa (AN) is a severe mental disorder characterized by significant weight loss or failure to gain weight appropriately for age due to restrictions of energy intake, an intense fear of weight gain, and a disturbed body image [1]. AN affects about 1.4% of young women and girls and 0.2% of boys and men at one point during their lifetime [2]. The typical onset age is between 14 and 22 years [3], and clinicians are thus often challenged with the decision of child- vs. adult-centered care, as many healthcare systems clearly distinguish between these services [4]. For patients treated at a transitional age, e.g., in late adolescence between ages 18 and 20, physicians and therapists may need to evaluate early treatment responses and take decisions with regard to the duration, intensity, and setting of treatment. Some clinicians prefer to base these decisions on fixed criteria, for example, a patient’s change in body mass index (BMI) and other measures of improved patient health [5]. However, this kind of evaluation requires an adequate understanding of how clinical data evolve throughout AN treatment, which data predict treatment trajectories and outcomes, and whether universal criteria apply across different groups of adolescents with AN.

Early treatment responses, especially weight gain during the first weeks, have been linked to improved AN treatment outcomes [6]. Early weight gain and fewer weight fluctuations have been linked to normalized discharge weight and improved psychopathology [7,8,9,10,11,12,13]. However, recent findings suggest that adolescents’ early weight changes might differ by patient gender [14], raising the question of whether evaluating young men’s and women’s clinical data during AN treatment may require different criteria.

Coelho et al. [15] observed stronger improvements in young men in terms of AN-related cognitions when comparing 20 young men (14 AN, 6 other) with 20 young women (12 AN, 1 BN, 7 other). Nagata et al. [16] witnessed that young men required a longer length of stay than young women and that they also exhibited greater weight change when comparing 95 young men (58 AN, 1 BN, 36 other) to 493 young women (363 AN, 8 BN, 1 BED, 121 other). Thus, although preliminary and not consistently replicated across all investigations [17], young men may respond more favorably during AN treatment than young women (for similar findings on adult patients, see, for example, Halbeisen et al. [18]). The predictive value of such early improvements for treatment outcomes by gender in adolescents remains unclear.

Using routine observational data of an inpatient cohort originally described elsewhere [18], the present study sought to provide further insights into the role of gender for adolescents’ early treatment responses and AN treatment outcomes. Specifically, we were interested in (a) whether young men and young women with AN differ in terms of admission psychopathology and early treatment response and, most importantly, (b) whether early treatment responses predict AN treatment outcomes similarly across genders. Based on the available data, early treatment responses were defined in terms of weight changes and weight variability (i.e., weight fluctuations around the trajectory of weight change) during the initial 14 days of treatment, which have specifically been linked to treatment outcomes among adolescents with AN [10].

Similar to findings described above [15,16], we previously observed within our inpatient cohort that (mostly adult) men with AN showed greater weight gains over the course of treatment compared to their (mostly adult) women counterparts, and that men with AN had greater improvements in terms of AN-related cognitions and general psychopathology [18]. However, our previous analysis considered age-, diagnosis-, and length-of-treatment-matched samples of men and women across all age groups, included mostly adult patients, and we neither specifically compared treatment parameters among adolescents nor investigated associations between early treatment responses and overall outcomes. By following a matched samples approach, the previous analysis also excluded the vast majority of available adolescent patient data, which served as the basis for the present analysis. It is thus still an open question whether adolescent men and women with AN differ in terms of early and overall treatment outcomes and to what extent early improvements may predict treatment outcomes similarly or differently across gender groups. Given the exploratory nature of our analyses, we refrained from making strong predictions.

## 2. Materials and Methods

### 2.1. Participants

We included datasets of adolescents of a larger cohort of 2158 individuals with eating disorders originally described elsewhere [18]. Specifically, we included all adolescents (patients aged 20 years or younger [19]) with anorexia nervosa (AN), who were admitted and treated for at least 14 days between January 2018 and December 2021 at the Klinik am Korso, Bad Oeynhausen, Germany. Inpatient treatment followed a multimodal rehabilitation concept based on psychodynamic and cognitive-behavioral approaches and included individual and group psychotherapy, psychoeducation, nutritional rehabilitation, and complementary therapies (e.g., body psychotherapy). Therapists were trained physicians and psychologists who participated in regular supervision.

The retrospective analyses were reviewed and approved by the Ethics Committee of the Ruhr-University Bochum’s Medical Faculty at Campus East-Westphalia as part of application AZ 2021-849. Datasets are available from the corresponding author upon reasonable request.

### 2.2. Materials and Procedure

Patient age and gender were recorded at admission (note that the sociodemographic question queried patients’ “Geschlecht”, which in German carries connotations for both assigned sex and gender). The nursing team measured height. Body weight (in kg), used for calculating BMI, was assessed using a calibrated scale (KERN & SOHN GmbH, Balingen-Frommern, Germany) at admission, daily during the first week after admission, and at least once per week as of week two of treatment. All individuals were asked to complete a battery of standardized assessments at admission and discharge, which we used to evaluate psychopathology and treatment outcomes.

ED-specific psychopathology at admission and discharge was assessed using the validated German version of the Eating Disorder Examination-Questionnaire (EDE-Q) [20,21,22]. The EDE-Q shows high levels of convergent validity [23], and assesses the occurrence of cognitive and behavioral symptoms of disordered eating within the last 28 days along four subscales, labelled “(Dietary) Restraint”, “Eating Concern”, “Shape Concern”, and “Weight Concern”, using 22 attitudinal items rated on a 7-point scale (from 1, never, to 7, every day; six additional items that assess the self-reported frequencies of weight control behaviors are not used for scoring). However, the proposed factor structure has received little support in previous studies, especially in men and mixed-gender samples [24,25]. Moreover, because the Restraint subscale queries eating behaviors that are not allowed under the clinic’s treatment regime, changes in this subscale are irrelevant for evaluating changes in psychopathology. We therefore only considered an average Concerns score (i.e., the mean of Eating, Shape, and Weight Concern) for the present analysis (Cronbach’s α = 0.89).

Body image concerns at admission and discharge were assessed using the validated Body Experience Questionnaire (Fragebogen zur Beurteilung des eigenen Körpers, FBeK) [26,27]. Its 52 dichotomous items (yes/no) assess Physical Attractiveness and Self-confidence (“I am attractive”; Cronbach’s α = 0.81), Accentuation of Physical Appearance (“The outer appearance says a lot about a person”; Cronbach’s α = 0.66), Insecurities and Concerns related to Appearance (“My appearance has already prevented me from connecting with others”; Cronbach’s α = 0.77), and Physical-sexual Discomfort (“I am satisfied with my sexual sensations” Cronbach’s α = 0.50). Gender-based percentile ranks of subscale means were provided for the present analysis.

The Symptom Checklist SCL-27+ [28,29] served as a validated brief measure of at-admission and at-discharge general psychopathology [30,31]. Twenty-seven items assess the presence of depressive (e.g., “melancholy”), vegetative (e.g., “heart palpitations”), agoraphobic (e.g., “becoming afraid of crowds”), and sociophobic symptoms (e.g., “feeling insecure when others look at me”) as well as pain (e.g., “chest pain”) over the last two weeks on a 5-point scale (from 1, never, to 5, very often). Further dichotomous questions address lifetime depressive symptoms and suicidality. However, as the proposed factorial validity received little empirical support [31], we only considered the overall score, the Global Severity Index (GSI; Cronbach’s α = 0.81), for the present analysis.

The Beck Depression Inventory (BDI) was also included as a widely used self-report inventory to assess the severity of depressive symptoms at admission and discharge [32]. The BDI contains 21 items, each scored on a 4-point scale, with sum scores ranging between 0 and 63 (Cronbach’s α = 0.87).

### 2.3. Data Aggregation and Statistical Analysis

For each patient, we computed admission and discharge scores for EDE-Q’s Concerns subscales, the BDI, and the SCL-27+ GSI, and the FBeK subscales, as described above. Weight outcomes were transformed to BMI-SDS (Standard Deviation Scores) using the LMS method [33] with patient admission height and German general population reference data [34,35]. BMI-SDS shows the deviation of a patient’s BMI from the population mean and allows for age- and gender-adjusted body weight comparisons. Similar to previous studies [10,36], we then computed daily weight change and weight variability within the first two weeks of treatment (starting with day 0 at admission and ending on day 13), using the slopes and root-mean-squared errors (RMSE) of residuals from a linear mixed model. The model used changes (increase or decrease) from baseline BMI-SDS to current BMI-SDS as dependent variable, and included an intercept and a fixed effect of day of treatments, with by-patient random variation of slope and intercept. The slopes of this model estimate, for each patient, the linear increase or decrease from admission BMI-SDS with each day of treatment (i.e., the Daily Weight Change) [10], and the RSME estimates the degree of variation of a patient’s weight around this regression line.

Questionnaire scores, Daily Weight Change, and RSME values were then used in two sets of analyses. First, we compared Daily Weight Change and RSME, along with basic information such as age, admission BMI-SDS, admission psychopathology scores, and total length of treatment, between adolescent men and women with AN using *t*-tests. Second, we conducted linear regression analyses to investigate whether Daily Weight Change and RSME predict discharge weight and psychopathology outcomes. These analyses included treatment duration and admission scores as covariates (mean-centered), as well as predictors for gender (1 = young men, 0 = young women) and its interaction with Daily Weight Change and RSME, respectively. This allowed us to compare for treatment outcomes between young men and women, and to estimate whether beneficial (or detrimental) effects of early treatment responses on treatment outcomes differ between the genders.

Descriptive results are reported as means, relative frequencies, and standard deviations (SDs). Effect sizes are reported as Cohen’s d. The significance level for all analyses was set at *p* ≤ 0.05. We applied Bonferroni correction for multiple comparisons on a per-questionnaire basis (i.e., for the FBeK subscales). We screened for and removed univariate outliers among questionnaire responses based on interquartile range (1st quartile–3 × interquartile range, 3rd quartile + 3 × interquartile range). *t*-tests and linear regressions were conducted using SPSS Statistics Version 28 for Windows [37]. Linear mixed models were fitted using restricted maximum likelihood (REML) estimation in R package lme4 Version 1.1.31 [38]. The R version used was 4.2.2 [39].

## 3. Results

**Figure 1:**
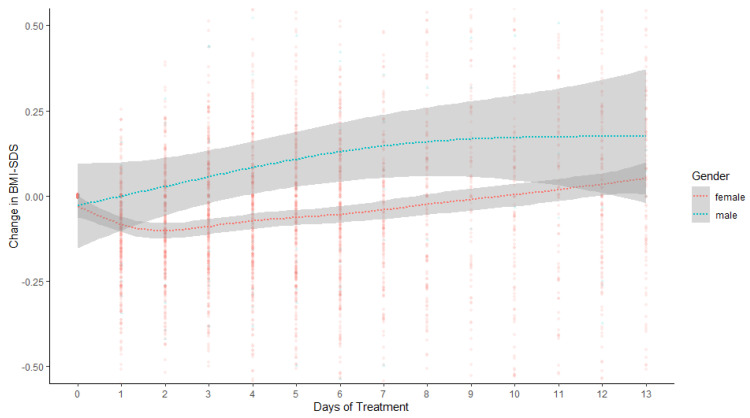
Patients’ weight changes, from baseline BMI-SDS to current BMI-SDS, as a function of gender, and timepoint. Curved regression lines (with 95% confidence bands) were fitted using function geom_smooth with method “loess” using R package ggplot2 Version 3.3.5 [40]. BMI-SDS = body mass index standard deviation score.

The total sample included 318 adolescent women with AN and 14 adolescent men with AN. Table 1 summarizes patient and treatment features and comparisons at admission, and Table 2 the admission vs. discharge comparisons. At admission, young men and women with AN were similar in age, BMI-SDS, and scores on the EDE-Q, FBeK, SCL-27+ GSI, and BDI. Treatment duration and weight RSME were also similar. However, significant differences in Daily Weight Change showed that young men gained weight faster early during treatment compared to young women (see Figure 1). From admission to discharge, all patients with AN gained in BMI-SDS and improved in terms of EDE-Q, SCL-27+ GSI, and BDI scores. Young women with AN also improved in three of the FBeK’s body image dimensions, whereas changes on the FBeK from admission to discharge in men with AN were not significant.

Table 3 shows the unstandardized weights of the regression analyses (bold values highlight significant weights at *p* ≤ 0.05). When adjusted for treatment duration and at-admission scores, adolescent men and women with AN remained overall comparable in terms of weight and psychopathology outcomes. However, Daily Weight Change positively predicted discharge BMI-SDS (i.e., lower early increase led to lower discharge weight). Lower levels of RSME predicted decreased EDE-Q Concerns and lower levels of FBeK body image Insecurities in both young men and women. Moreover, weight gain predicted higher general SCL-27+ GSI psychopathology scores at discharge, but only among young men, as indicated by the significant Daily Weight Change × Gender interaction. At the same time, lower RSME predicted higher SCL-27+ GSI scores at discharge for men, which could indicate that constant weight gain (with low variability) may incur additional psychological burden for young men.

## 4. Discussion

The typical onset age of AN [3] creates challenges for physicians and therapists that may need to evaluate early treatment responses and decide about the duration and intensity of treatment, which may differ between children- and adult-centered services. An adequate understanding of which clinical measures may predict treatment trajectories and outcomes in young men and women may aid such decision processes. Here, we investigated gender differences with regard to the predictive value of early weight changes and weight variability for weight- and psychopathology-related outcomes of adolescents with AN. At admission, we found that eating disorder-related as well as general psychopathology scores were similar in young men and young women with AN. Also, all assessed parameters improved during AN treatment in a similar manner for both young men and women—with the exception that young men showed no significant improvements in measures of body image concerns. The lack of improvement in body image in young men could be due to young men being less dissatisfied with their body to begin with or to more treatment-resistant body image concerns in young men. Alternatively, body image concerns and their improvement in young men may be not adequately captured by the currently used assessments which were mostly developed to assess symptoms in women [41].

With regard to daily weight changes, we found that young men with AN gained weight faster than young women with AN, and that daily weight changes predicted patient discharge weight across all gender groups. Low weight variability was also predictive of lower disordered eating psychopathology and lower body image insecurities at discharge, similar to previous observations in patients with bulimia nervosa [42]. This could be explained if weight variability were to be considered the expression of conscious efforts to control or delay weight gain. Thus, low weight variability may be considered the expression of a less severe AN-related psychopathology. However, we also observed that lower weight gain predicted lower general psychopathology scores for young men with AN, whereas lower weight variability was predictive of higher general psychopathology scores in the same group at discharge. Thus, there is a discrepancy between weight variability and AN-related vs. general psychopathology outcomes in young men only. While one would expect changes in AN-related and general psychopathology to go hand in hand, our findings might indicate that this is not the case in young men. This could suggest an increased risk for psychological burden among young men with AN experiencing body changes, especially when they were gaining weight constantly (note that the statistical model estimates effects of weight variability while controlling for daily weight changes). Given the lack of data on further behaviors during early treatment in our sample, further explorations of antecedents and consequences of weight variability in AN are needed.

It is important to note that the effects of weight fluctuations, as measured by the degree of variation around a patient’s weight regression line, have thus far only been investigated for AN in two studies, with varying results. Whereas Kolar et al. [10] found no effects of weight variability on treatment outcomes in adolescent and adult patients, higher weight variability predicted poorer weight gain in adults in Hartmann et al. [11]. Besides the difference in sample choice, neither study analyzed effects of weight variability stratified by gender; this might be a further explanation for the reported discrepancies between studies and highlights the need for further investigations.

Taken together, the present findings suggest that early weight changes and variability are similarly important predictors of treatment outcomes in adolescent men and women with AN but may also hint at possible gender differences in terms of general psychopathology and specific body image concerns. Faster weight improvements observed here and in other studies among young men with AN might indicate that shorter treatment durations may be required (or tolerated). It is important to acknowledge, however, that young men remain underrepresented in eating disorders research and care [41,43], and that these findings may well reflect a biased selection of adolescent men going into treatment. Clearly, more research is needed to corroborate these observations.

Certainly, we must note other limitations as well. Men remain underrepresented in eating disorders research, and in studies on AN specifically, necessitating less reliable small-with-large sample comparisons in need of further replication. Moreover, with data collected exclusively at an eating disorders specialty clinic, evidence for gender parity could be limited to the more severe cases admitted to inpatient treatment, or to treatment settings with high levels of expertise and experience. We also only report on immediate treatment responses, raising the question of whether long-term outcomes still remain comparable among gender groups. Finally, we must note that there are several indications that currently used diagnostic criteria and psychopathology assessment tools (such as the EDE-Q) may be suboptimal in capturing AN psychopathology in men [25,44], which may have artificially homogenized our sample, and/or precluded the assessment of further gender differences.

## 5. Conclusions

The present findings suggest that early weight changes and variability are similarly important predictors of weight gain and psychopathology outcomes in adolescent men and women with AN, with possible gender differences in terms of the prediction of general psychopathology. Future studies will need to further explore and corroborate to what extent early clinical improvements predict treatment outcomes for adolescents with AN across genders.

## Figures and Tables

**Table 1:** Patients with AN admission and treatment characteristics and gender comparisons.

Variable	Gender	*n*	M	SD	d
age (years)	young men	14	17.1	2.1	0.05 [−0.49; 0.58]
	young women	318	17.1	1.9	
BMI-SDS	young men	14	−2.2	1.7	0.17 [−0.37; 0.70]
	young women	318	−2.4	1.4	
treatment length	young men	14	52.1	20.2	−0.46 [−0.99; 0.08]
	young women	318	60.9	19.2	
Weight Change	young men	14	0.03	0.06	0.85 * [0.31; 1.38]
	young women	318	0.00	0.03	
RSME	young men	14	0.10	0.05	0.31 [−0.23; 0.85]
	young women	318	0.09	0.06	
EDE-Q Concerns	young men	13	4.5	1.5	−0.27 [−0.82; 0.29]
	young women	293	4.9	1.2	
FBeK Attract.	young men	12	3.1	3.8	−0.01 [−0.59; 0.56]
	young women	271	3.1	2.9	
FBeK Accent.	young men	13	84.3	17.4	0.18 [−0.38; 0.74]
	young women	293	80.6	20.4	
FBeK Insecur.	young men	13	88.5	11.1	0.2 [−0.36; 0.76]
	young women	280	85.2	16.2	
FBeK Disc.	young men	13	90.7	13.4	0.42 [−0.14; 0.97]
	young women	293	82.4	20.0	
SCL GSI	young men	14	1.9	0.6	−0.02 [−0.56; 0.52]
	young women	299	1.9	0.7	
BDI	young men	14	27.6	10.8	0.02 [−0.51; 0.56]
	young women	304	27.3	10.9	

Note. BMI-SDS = body mass index (kg/m^2^) standard deviation scores, RSME = root-mean-squared errors of weight prediction residuals, EDE-Q = Eating Disorder Examination-Questionnaire, FBeK = Body Experience Questionnaire (Fragebogen zur Beurteilung des eigenen Körpers), Attract. = Attractiveness, Accent. = Accentuation, Insecur. = Insecurity, Disc. = Physical-sexual Discomfort, SCL GSI = symptom check list global severity index, BDI = Beck Depression Inventory. Cohen’s d-values marked with an asterisk highlight significant differences at *p* ≤ 0.05 (after Bonferroni adjustment, where applicable).

**Table 2:** Cohen’s d with 95% confidence interval (in parentheses) for pre–post (admission–discharge) comparisons of AN patients by gender.

Comp. (Pre–Post)	Young Men	Young Women
BMI-SDS	−0.65 * [−1.21; −0.06]	−0.69 * [−0.81; −0.56]
EDE-Q	1.45 * [0.34; 2.52]	1.23 * [1.06; 1.39]
FBeK		
Attract.	−0.70 [−1.51; 0.16]	−0.49 * [−0.63; −0.35]
Accent.	−0.05 [−0.79; 0.69]	−0.33 * [−0.46; −0.20]
Insecur.	0.45 [−0.30; 1.16]	0.29 * [0.16; 0.41]
Disc.	0.48 [−0.27; 1.20]	0.16 [0.03; 0.28]
SCL GSI	2.22 * [0.87; 3.53]	0.56 * [0.43; 0.69]
BDI	1.98 * [0.73; 3.18]	1.06 * [0.91; 1.21]

Note. BMI-SDS = body mass index (kg/m^2^) standard deviation scores, EDE-Q = Eating Disorder Examination-Questionnaire, FBeK = Body Experience Questionnaire (Fragebogen zur Beurteilung des eigenen Körpers), Attract. = Attractiveness, Accent. = Accentuation, Insecur. = Insecurity, Disc. = Physical-sexual Discomfort, SCL GSI = symptom check list global severity index, BDI = Beck Depression Inventory. Cohen’s d-values marked with an asterisk highlight significant differences at *p* ≤ 0.05 (after Bonferroni adjustment, where applicable).

**Table 3:** Unstandardized weights of the regression analyses on discharge weight and psychological outcomes of patients with AN (standard errors in parentheses).

Predictors	BMI-SDS	EDE-Q	FBeK Attract.	FBeK Accent.	FBeK Insecur.	FBeK Disc.	SCL GSI	BDI
Intercept	**−1.87** **(0.03)**	**3.38** **(0.07)**	**7.76** **(0.62)**	**87.23** **(0.94)**	**78.85** **(1.30)**	**78.66** **(1.26)**	**1.60** **(0.03)**	**16.84** **(0.52)**
Gender (0 = women, 1 = men)	0.06 (0.15)	−0.65 (0.55)	1.73 (5.60)	−18.91 (9.52)	−9.01 (10.01)	−5.72 (9.99)	**−0.54** **(0.22)**	−6.49 (4.22)
Treatment Duration	**0.01** **(0.00)**	0.00 (0.00)	−0.05 (0.03)	−0.03 (0.05)	0.04 (0.07)	0.09 (0.07)	0.00 (0.00)	0.04 (0.03)
Daily Weight Change	**13.67** **(1.00)**	**−2.53** **(2.44)**	2.80 (21.71)	21.87 (33.50)	1.34 (45.72)	75.78 (45.01)	0.16 (1.00)	−5.76 (18.80)
RSME	0.58 (0.54)	**2.55** **(1.18)**	−10.28 (10.26)	40.91 (16.40)	**64.26** **(22.13)**	11.39 (21.91)	−0.14 (0.48)	−9.22 (9.23)
Daily Weight Change × Gender	−4.22 (4.16)	11.66 (18.67)	−86.03 (141.01)	285.34 (246.45)	320.68 (278.14)	305.94 (276.76)	**12.74** **(6.13)**	121.58 (117.26)
RSME × Gender	−2.77 (4.55)	−19.32 (16.76)	224.73 (126.47)	−246.94 (221.17)	−475.81 (262.31)	−571.94 (261.24)	**−14.59** **(5.79)**	−144.04 (110.96)
Score at Admission	**0.85** **(0.02)**	**0.58** **(0.06)**	**1.26** **(0.21)**	**0.41** **(0.05)**	**0.69** **(0.08)**	**0.55** **(0.06)**	**0.68** **(0.04)**	**0.58** **(0.05)**

Note. BMI-SDS = body mass index (kg/m^2^) standard deviation scores, RSME = root-mean-squared errors of weight prediction residuals, EDE-Q = Eating Disorder Examination-Questionnaire, FBeK = Body Experience Questionnaire (Fragebogen zur Beurteilung des eigenen Körpers), Attract. = Attractiveness, Accent. = Accentuation, Insecur. = Insecurity, Disc. = Physical-sexual Discomfort, SCL GSI = symptom check list global severity index, BDI = Beck Depression Inventory. Bold values highlight significant differences at *p* ≤ 0.05 (Bonferroni-adjusted, where applicable).

## Data Availability

The data presented in this study are available upon reasonable request from the corresponding author.
